# Seizure prediction in stroke survivors who experienced an infection at skilled nursing facilities—a machine learning approach

**DOI:** 10.3389/fphys.2024.1399374

**Published:** 2024-05-30

**Authors:** Madeleine Stanik, Zachary Hass, Nan Kong

**Affiliations:** ^1^ Purdue University, Department of Engineering, Weldon School of Biomedical Engineering, West Lafayette, IN, United States; ^2^ Purdue University, Schools of Industrial Engineering and Nursing, West Lafayette, IN, United States

**Keywords:** stroke, seizure, infection, machine learning, binary classification, minimum data set, skilled nursing facility

## Abstract

**Background:**

Infections and seizures are some of the most common complications in stroke survivors. Infections are the most common risk factor for seizures and stroke survivors that experience an infection are at greater risk of experiencing seizures. A predictive model to determine which stroke survivors are at the greatest risk for a seizure after an infection can be used to help providers focus on prevention of seizures in higher risk residents that experience an infection.

**Methods:**

A predictive model was generated from a retrospective study of the Long-Term Care Minimum Data Set (MDS) 3.0 (2014–2018, n = 262,301). Techniques included three data balancing methods (SMOTE for up sampling, ENN for down sampling, and SMOTEENN for up and down sampling) and three feature selection methods (LASSO, Recursive Feature Elimination, and Principal Component Analysis). One balancing and one feature selection technique was applied, and the resulting dataset was then trained on four machine learning models (Logistic Regression, Random Forest, XGBoost, and Neural Network). Model performance was evaluated with AUC and accuracy, and interpretation used SHapley Additive exPlanations.

**Results:**

Using data balancing methods improved the prediction performances of the machine learning models, but feature selection did not remove any features and did not affect performance. With all models having a high accuracy (76.5%–99.9%), interpretation on all four models yielded the most holistic view. SHAP values indicated that therapy (speech, physical, occupational, and respiratory), independence (activities of daily living for walking, mobility, eating, dressing, and toilet use), and mood (severity score, anti-anxiety medications, antidepressants, and antipsychotics) features contributed the most. Meaning, stroke survivors who received fewer therapy hours, were less independent, had a worse overall mood were at a greater risk of having a seizure after an infection.

**Conclusion:**

The development of a tool to predict seizure following an infection in stroke survivors can be interpreted by providers to guide treatment and prevent complications long term. This promotes individualized treatment plans that can increase the quality of resident care.

## 1 Introduction

For the past decade, stroke has ranked in the top five leading causes of death in the United States (US) (Ahmad and Anderson, 2021; Heron, 2021; Shiels et al., 2022). Stroke related deaths account for 4.7% of deaths across all age groups and 6.1% of deaths in aging populations classified as age 65 and older (Shiels et al., 2022). However, not all strokes are fatal and 60% of ischemic stroke patients and 38% of hemorrhage stroke patients survive the first year (Smajlović et al., 2006). Patients that survive often face serious complications or disabilities. In fact, stroke is the leading cause of serious long-term disability in the United States and each year accounts for about $56.5 billion dollars (CDC, 2023).

Within the last few years, the number of stroke related deaths has been decreasing (Chohan et al., 2019). With this increased survival rate, there has been an increase in the number of patients with complications. The major complications include recurrent stroke (9% of patients), epileptic seizure (3%), urinary tract infection (24%), chest infection (22%), other infections (19%), falls (25%), shoulder pain (9%), other pain (34%), depression (16%), anxiety (14%), emotionalism (12%), and confusion (56%) (Langhorne et al., 2000). By focusing on the prevention of these complications, the long-term survival rate and quality of life for stroke survivors can be improved.

It has been well documented that infections are a leading risk factor for seizures and epilepsy (Vezzani et al., 2015). However, there has not been extensive research into how infections impact seizure risk in stroke survivors. Stroke survivors are especially prone to both bacterial and viral infections, and having these infections may consequently increase their seizure risk (Langhorne et al., 2000). Having frequent infections and seizures could severely postpone the patient’s recovery process and possibly result in death. Exploring this coupling of complications could help prevent adverse effects by placing a stronger emphasis on limiting infection and preventing seizure in patients who have already had an infection.

To help prevent infection and subsequent seizure, focusing on the patient’s recovery through their rehabilitation plan is a promising pathway. When a stroke survivor is discharged from the hospital or other treatment facilities to a skilled nursing facility (SNF), they will begin rehabilitation following a set plan (Bindawas and Vennu, 2016). The effectiveness of this set plan at the SNF relies heavily on the team of professionals that goes into making it (Lenze et al., 2012). In fact, it has been shown that rehabilitation plans made by a group of professionals are more effective than those made by a single professional (Graham, 2013). In addition, if the team takes the time to specialize the plan, it has been shown that the patient will have a faster recovery rate and yield better functional outcomes (Bindawas and Vennu, 2016). Other studies have also shown that specialized plans yield greater participant engagement with activities being completed at higher intensities (Lenze et al., 2012). These specialized rehabilitation plans are typically variations of a standardized version and vary depending on the patient’s severity of complications and response to the therapy (Bernhardt et al., 2016). However, plans are adjusted by healthcare professionals using intuition rather than numerical feedback, which leads to plans that fail to help patients reach their recovery goal (Levinson, 2013). If the plans were individualized and a patient’s response to changes in the plan could be measured with concrete numerical evidence, then the outcome of recovery could improve for stroke survivors.

Additionally, it has been shown that stroke survivors at nursing facilities receive fewer hours of rehabilitation compared to hospital settings (Koopmans et al., 2010). This is typically a result of the reduction in staffing and intensity of care, but receiving more therapy hours has been associated with increased independence (Jette et al., 2005), greater likelihood of discharge from SNF to community (Jette et al., 2004; Jung et al., 2016), and greater functional improvements (Chen et al., 2002). This means that residents at SNFs could benefit from an increase in therapy hours as part of their rehabilitation. With stroke survivor rehabilitation plans typically lasting between a few months to a few years (Bindawas and Vennu, 2016), this is considered long-term rehabilitation (IHCP, 2023). Assessing the relationship between the number of therapy hours in a rehabilitation plan and the risk of seizure following an infection could yield beneficial results in resident recovery.

This study used the Long-Term Care Minimum Data Set (MDS) 3.0 (2014–2018) in a midwestern US state to retrospectively investigate the risk of seizure following an infection both short term and long term. By focusing on the stroke to infection to seizure pathway, this study seeks to identify risk factors for seizure after an infection to then help limit seizures in stroke survivors who have experienced an infection. The model is fit to predict the risk, return an individualized resident risk estimate, and interpret which factors contribute the most to this risk estimate. Uncovering which factors contribute the most to seizure risk may aid healthcare professionals in adjusting rehabilitation plans to improve resident outcomes.

Other studies have predicted the risk of seizure in stroke surviving patients (Bunney et al., 2022; Looti et al., 2023; Lekoubou et al., 2024); however, none exist that include the infection to seizure pathways in stroke survivors. Another novel aspect is the use of the MDS data set for prediction of seizures in stroke survivors, which has not even been used for seizure prediction. The third novel aspect of this study is the use of SHapley Additive exPlanations (SHAP) for model interpretations, and though this technique was developed a number of years ago, its application to the healthcare space is relatively novel.

## 2 Materials and methods

This study had two specific aims for investigating the risk of seizure following infection in stroke survivors at skilled nursing facilities (SNFs).

1. Determine the risk ratio of stroke survivors experiencing a seizure after an infection short term (within 14 days) and long term (within 1 year).
2. Interpret the resultant predictive models to identify risk factors for a stroke surviving nursing home resident experiencing a seizure within 14 days following an infection.

The data initially includes all individuals admitted to a Medicare and Medicaid licensed SNF between 1 January 2014 to 20 April 2018 in Indiana taken from the Long Term Care Minimum Data Set. All assessments during the time period were evaluated. The main data features include demographic, diagnosis, activities of daily living (ADL), pain, treatment, mobility, and therapy. Residents with a previous history of seizure and epilepsy disorder were excluded in order to establish the temporal association between stroke and seizure occurrence.

### 2.1 Risk ratio

Prior to modeling, a preliminary analysis was conducted to verify the relationship between stroke survivors, infections, and seizures. Stroke survivors considered were nursing facility residents with the stroke diagnosis code who remained in a skilled nursing facility (SNF) after the incident. This included both residents admitted with a stroke diagnosis and those who had a stroke while in the SNF. An infection was said to have occurred if any of the urinary tract infection, pneumonia, sepsis, tuberculosis, viral hepatitis, wound infection, and multidrug resistant organism diagnosis codes were noted in an assessment after the stroke noted assessment. A seizure was said to occur if the diagnosis code for seizure and epilepsy disorder was noted in an assessment after the infection noted assessment.

Assessments from stroke survivors were used to count the number of unique residents for four mutually exclusive categories. Divisions were based on the occurrence of an infection and/or stroke. The initial data were first split on the occurrence of stroke in resident assessments, which yielded 24,570 stroke survivor residents. The data was then split on whether a resident had an infection within 75 days following the stroke, a time period associated with increased risk of disability and death (Finlayson et al., 2011; Ulm et al., 2012; Learoyd et al., 2017). It was found that 2,861 unique residents had an infection within 75 days following a stroke. These could have been a resident who had a stroke in the SNF and then had an infection within 75 days following, or a resident who was admitted to the SNF with the stroke diagnosis and had an infection within 75 days from their first assessment. For the latter, this means the resident entered the facility with the stroke diagnosis and the infection threshold was set to within 75 days from the first assessment date. An additional 21,709 residents did not have an infection following a stroke.

**FIGURE 1:**
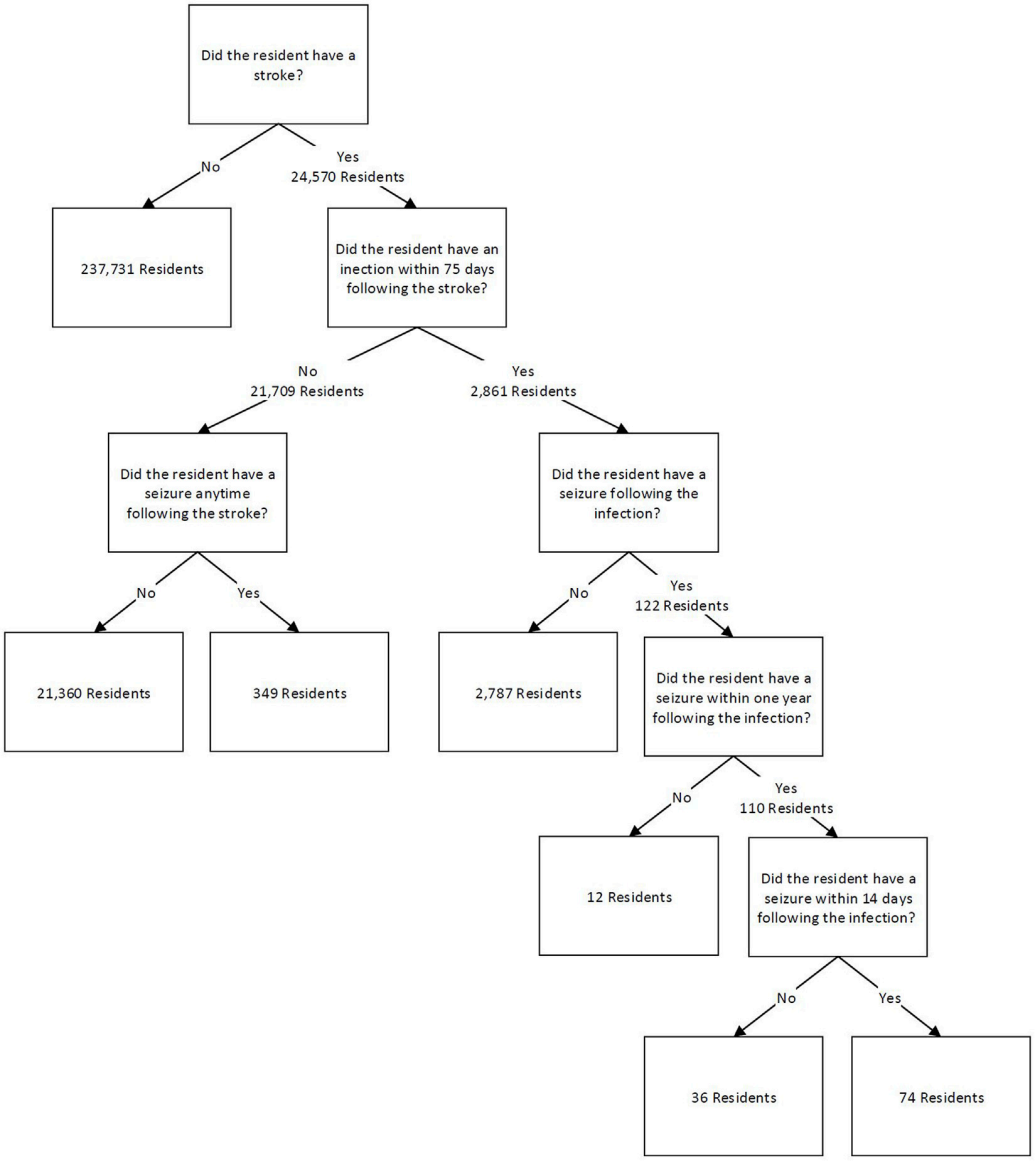
Resident categorization flow chart.

These two groups were each split into two groups based on whether residents had a seizure following their stroke. The next two categories are sub-divisions of the first category based on the timing of the appearance of the seizure diagnosis. For the stroke survivors that had an infection within 75 days following their stroke, it was further evaluated if the resident had a seizure anytime following the infection or within 14 days following the infection. This value of 14 days was obtained from a study that found that seizures usually occur within one to 2 weeks following an infection, so 14 days was chosen based on the 2-week mark (Vezzani et al., 2015). Other studies have also found that seizures can occur after a stroke over 5 years later (Naess et al., 2004; Myint et al., 2006), so a long-term value after infection was also assessed for comparison as part of this risk ratio. For this study, this value was 1 year after the infection. The long-term follow-up period of 1 year had 110 residents experience a seizure following an infection, and the short term, 14 day follow up period, had 74 residents experience a seizure following an infection. For stroke survivors that did not experience an infection, it was determined that 349 residents had a seizure any amount of time following a stroke with no reported infection prior to the seizure. Figure 1 demonstrates these groups and their breakdown as a flow chart.

Residents admitted near the end of the dataset were removed if they did not experience a post-infection seizure and there was not an adequate number of days to observe the full follow-up period (right censoring of data). As an example, for 14-day post-infection seizure, a resident who had only 12 days of follow up in the data, but experienced an infection then had a seizure during that follow-up was kept in the data. A resident with only 12 days of follow up who did not have a seizure before the end of the dataset, however, was removed due to right censoring of the data. For 14-day follow-up, 135 residents were right censored, and for 1-year follow-up, 867 residents were right censored. Residents were also right censored following the same method for the 75-day follow-up period between stroke and infection. This latter group had 6,051 residents with right censoring of their data. These censored residents were subtracted from risk ratio calculations and were removed from the predictive models.

The categorized residents and their corresponding prevalence were used to calculate risk ratios based on the number of unique residents who experienced a seizure. Using unique residents reduced the possibility of carrying forward diagnoses in the data between assessments that could have artificially inflated occurrences. Therefore, the number of unique residents is a more robust method compared to the number of occurrences for calculating the risk ratio here. For a more detailed explanation, please see the discussion section. The ratios in Table 1 indicate that having an infection within 75 days after a stroke increases a resident’s risk of having a seizure within 14 days post infection by 1.20-fold. Having an infection within 75 days after a stroke increases a resident’s risk of having a seizure 1 year post infection by 2.42-fold. Risk ratios were calculated by comparing the population of individuals who experienced a seizure following an infection to those who experienced a seizure without first experiencing an infection (Table 2). The 95% confidence intervals did not contain one for the risk ratio of the 1-year follow-up period, indicating that the relative risk ratio was found to be statistically significant. This is consistent with current literature that indicates that infections increase the risk of seizures (Langhorne et al., 2000). The 14-day follow-up period’s 95% confidence interval for the risk ratio did contain 1, so this risk ratio was not found to be statistically significant. However, a large proportion of the post-infection seizures occurred within this time frame and adjustment for additional features can be informative, so modeling was also completed for prediction of seizure over the 14-day follow-up period.

**TABLE 1:** Risk ratio of seizure in stroke survivors with infections.

In Stroke Survivors	Relative Risk Ratio	95% Confidence Interval
Experiencing a seizure within 14 days after an infection	1.1968	[0.9344, 1.5330]
Experiencing a seizure within a year after an infection	2.4168	[1.9604, 2.9795]

**TABLE 2:** Risk ratio calculations of seizure in stroke survivors with infections.

In Stroke Survivors	Relative Risk Ratio	95% Confidence Interval
Experiencing a seizure within 14 days after an infection	$\frac{\frac{74}{\left(74+2787+12+36-135\right)}}{\frac{349}{\left(349+21360-6051\right)}}$	$e^{\ln\left(1.5832\right)\pm 1.96\sqrt{\frac{1}{74}+\frac{1}{2700}+\frac{1}{349}+\frac{1}{15309}}}$
Experiencing a seizure within a year after an infection	$\frac{\frac{110}{\left(110+2787+12-867\right)}}{\frac{349}{\left(349+21360-6051\right)}}$	$e^{\ln\left(2.6525\right)\pm 1.96\sqrt{\frac{1}{110}+\frac{1}{1932}+\frac{1}{349}+\frac{1}{15309}}}$

### 2.2 Data processing and modeling

The MDS data collection instrument includes 23 sections that contain information such as demographics, diagnoses, independence in performing activities of daily living, mood assessment, therapy, and medications by class. Each section contains data on all residents, and most residents had multiple entries in the dataset. These multiple entries were a result of periodic assessments (e.g., 5-day, 14-day, 30-day, 60-day, or 90-day post admission for Medicare Part A stays; admission, quarterly, and significant change in status for other stay types) that varied by resident when information would be updated. The date of the assessment was noted, and a de-identified person number was used to associate residents to all their assessments. The data was structured with the same number of rows appearing across all sections, but each section had a variable number of columns. The rows for each section match up directly by row index, so any row across all sections were the same assessment for the same resident. The columns were different sets of features broken up by sections, and within each section columns were related. For example, the therapy section contains columns for speech, occupational, physical, and other types of therapy. For this analysis, 149 features were selected from the thousands of features across the 23 sections.

These features included demographics (age, gender, marital status, race, height, and weight), treatments (physical therapy, occupational therapy, speech therapy, recreational therapy, psychological therapy, and medications), physical condition (daily activities, mobility, balance), and behavior (mood, pain, and delirium). These were the main feature groups, and all features were composed of more specific subgroups within these main groups. For example, in the category of physical therapy, there were variables on weekly individual minutes, concurrent minutes, group minutes, and number of days of therapy per week. The selection of these features followed other stroke survivor outcomes studies (Kelly-Hayes et al., 1998; Gittins et al., 2020).

Features with more than 70% missing values were removed (29 features removed). With 149 features to start, removing these 29 features reduced the total to 120. The remaining missing values were imputed using a two-step process. First, the resident’s most recent value from a prior or future assessment was carried forward or backwards. For example, if age was missing but a resident’s record from the previous month contained their age to be 65, the missing record was filled in with 65. For some features, no records were present for any entries, so as the second step, these remaining missing values were imputed with the k nearest neighbors method using five nearest neighbors. Missing values in diagnosis codes such as stroke, seizure, and infection were imputed with a zero indicating that event did not occur to prevent possible misdiagnosis or error carried forward. Dropping features with 70% missingness and using kth nearest neighbors with five neighbors is relatively common in healthcare datasets where missingness is relatively high (Wells et al., 2013; Salgado et al., 2016; Jäger et al., 2021). The use of 70% is on the higher end of what is found in the literature but was used as a matter of practicality. If the missingness cut off is set too low, a large proportion of data will be removed. Imputation using the resident’s most recent value from another assessment and imputation of diagnostics with zeros was author determined. Imputation using the resident’s other assessment could cause slight discrepancies, such as when imputing age, the method does not consider the resident’s birthday (data element not available for this work), but the imputed value is still likely to be very near the true value and resident specific.

Following imputation of missing data, the data was balanced using three methods. These methods were the Synthetic Minority Oversampling Technique (SMOTE) for up sampling, Edited Nearest Neighbor (ENN) for down sampling, and SMOTEENN for up and down sampling. Applying each balancing technique resulted in three sets of balanced data that then underwent three feature selection methods: Least Absolute Shrinkage and Selection Operator (LASSO), Recursive Feature Elimination (RFE), and Principal Component Analysis (PCA). These methods were selected from other studies that aimed to predict seizures post-stroke (Bunney et al., 2022; Looti et al., 2023; Lekoubou et al., 2024). These studies did not consider infections post-stroke; however, incorporation of post-stroke infections is not expected to significantly impact the results of feature selection methods. A balancing and feature selection technique was then chosen to apply to four different modeling methods: Logistic Regression, Random Forest, XGBoost, and Neural Network. Logistic regression was chosen for its distinction as one of the most fundamental modeling methods due to its linearity assumption, low computational intensity, and parametric interpretability. XGBoost and Random Forest were chosen due to their non-linear nature and ability to guard against underfitting and overfitting respectively. Neural Network was chosen because it is also non-linear and is not a tree-based model making for more interesting model comparisons and it has a strong ability to handle more complex relationships.

Hyper parameters for Logistic Regression (penalty: L1, L2 and C: 0.01, 0.1, 1, 10), XGBoost (learning rate: 0.1, 0.01, 0.001; and maximum depth: 1, 5, 10, 20), Random Forest (maximum depth: 1, 5, 10, 20; and n estimators: 200, 1,000, 10,000), and Neural Network (maximum iterations: 100, 200; activation layer: logistic, tanh; and number of hidden layers: 2, 8, 64, 128) were tuned using GridSearchCV. This method used all combinations of hyperparameters within each model then chooses the one with the best specified metric, which in this case was ROC and AUC. For example, the Logistic Regression method tested a total of six different models and chose the hyperparameters that yielded the greatest AUC of the six. For Logistic Regression, the selected hyperparameters were a penalty of L2 and a C of 0.1. The XGBoost model yielded 0.1 for the learning rate and 10 for maximum depth. Random Forest resulted in 10 for maximum depth and 1,000 for n estimators. Lastly, Neural Network chose 100 for maximum iterations, tanh for the activation layer, and 64 for the number of hidden layers.

Model performance was evaluated using prediction metrics such as Receiver Operator Curve Area Under the Curve (AUC), accuracy, recall, true positive rate, true negative rate, sensitivity, specificity, positive predictive values, negative predictive values, and precision. Data was split 80% and 20% for the training and testing set. Within the training set, 5-fold validation was used for tuning hyperparameters. The testing set was used to evaluate the model performance and interpretation. Model interpretation was evaluated with SHapley Additive exPlanations (SHAP).

## 3 Results

### 3.1 Demographics

**FIGURE 2:**
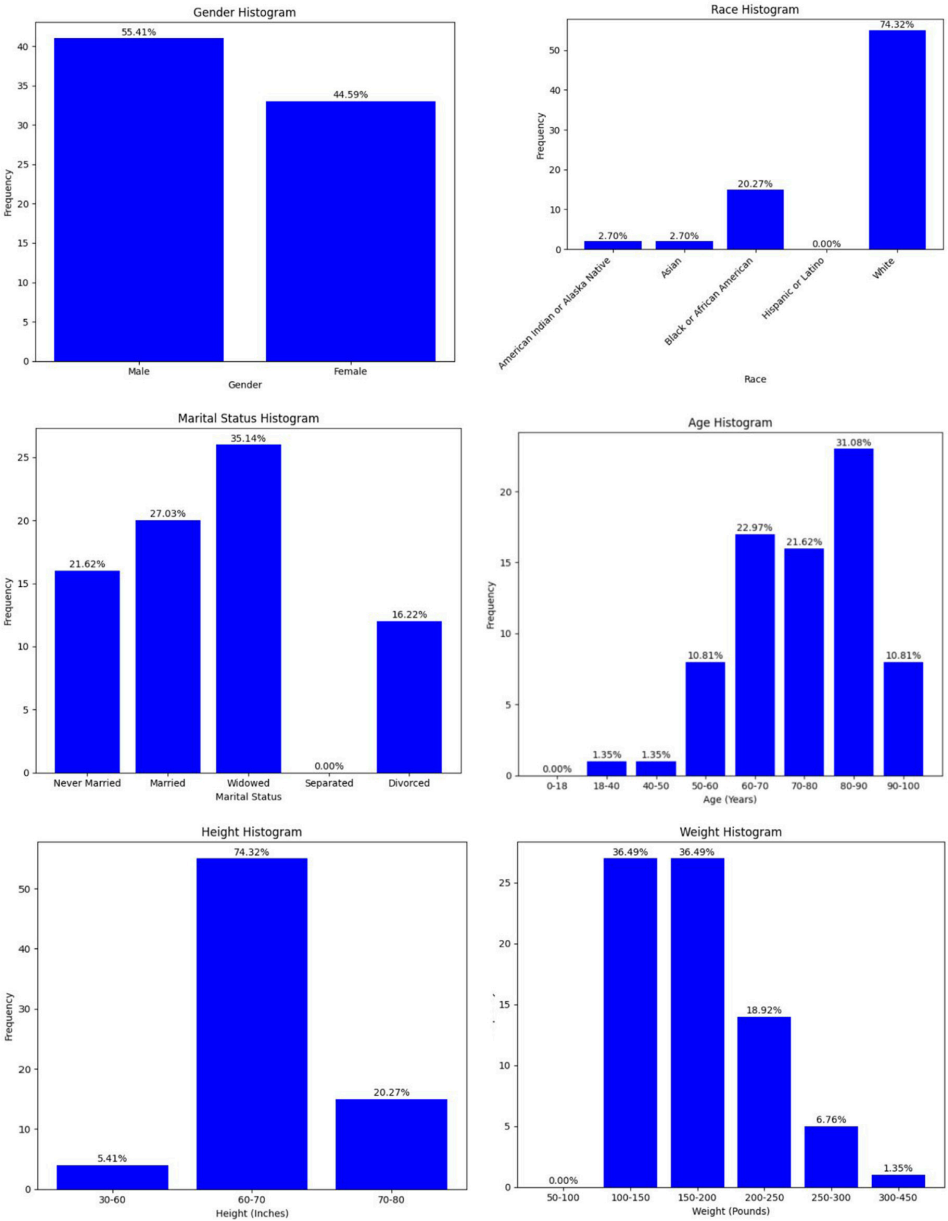
Demographic breakdown for fourteen-day risk.

Figure 2 shows the demographic breakdown of residents who suffered a seizure within 14 days post infection. From these demographics, older residents were more prevalent. The figure also shows that post-infection seizures were more prevalent in men, despite the MDS dataset containing primarily female residents. For the other demographic features, the trend follows those of the overall MDS dataset, so they are not as significant.

### 3.2 Model

The selected models used SMOTEENN for data balancing and all four models (Logistic Regression, XGBoost, Random Forest, and Neural Network) were assessed for prediction quality and feature interpretation. The data balancing method was chosen based on the breakdown of the classes. For ENN, the distribution of no seizures after infection to seizures following infection was 99% and 1%, which was likely the result of highly imbalanced data that interrupts the methodology of ENN and did not allow for down sampling. For SMOTE, the distribution was 49% and 51%, and for SMOTEENN the distribution was 50% and 50%. Because SMOTEENN yielded the most equal distribution, this data balancing method was selected.

For feature selection, PCA was adjusted to a range of component numbers and used the same set of features as the other methods, but this method was not selected since it did not allow for the same degree of interpretability. For RFE, results suggested that no features needed to be removed from the model. RFE worked by fitting a model with all the features then ranked each feature depending on the contribution to the model. Features were then removed based on if they meaningfully contributed to the scoring metric, which was Area Under the Receiver Operator Curve (AUC) for this study. Results indicated no features were to be removed so all features meaningfully contributed to the AUC. For LASSO, 17 features were suggested to be removed from the model. The LASSO method worked by assigning a coefficient to each feature based on its contribution to the model then shrinking the coefficients using the selected regularization parameter alpha, in this case alpha was 0.00001. A cut off was set of 0.001, meaning any features with a coefficient smaller than this value (features whose coefficient shrunk to zero) would be removed. If no coefficients were reduced to zero, then no features were removed. However, we took the conservative approach of siding with RFE which gauged all features as important keeping all features in the models.

For modeling methods, Logistic Regression, XGBoost, Neural Network, and Random Forest all yielded accurate prediction results (Table 3). Overfitting was assessed through K-fold cross validation with five folds on the training set, and the result of the cross validation returned five scores also close in value and confirmed that these models were not overfit (Table 4). However, the high accuracy generated by XGBoost, Neural Network, and Random Forest may have been a result of data balancing, where up sampling created more distinct entries that were easier to predict.

**TABLE 3:** Model performance metrics for fourteen-day risk prediction.

Parameter	Logistic Regression	XGBoost	Neural Network	Random Forest
AUC	0.8380	0.9999	0.9988	0.9999
Accuracy	0.7654	0.9999	0.9991	0.9998
Recall	0.7838	0.9999	0.9999	0.9997
True Positive Rate (TPR)	0.7838	0.9999	0.9999	0.9997
True Negative Rate (TNR)	0.7468	1.0000	0.9981	1.0000
Sensitivity	0.7838	0.9999	0.9999	0.9997
Specificity	0.7468	1.0000	0.9981	1.0000
Positive Predictive Values (PPV)	0.7571	1.0000	0.9981	1.0000
Negative Predictive Values (NPV)	0.7743	0.9999	0.9999	0.9997
Precision	0.7571	1.0000	0.9981	1.0000

**TABLE 4:** K fold cross validation scores for fourteen-day risk prediction.

Cross Validation Scores	Logistic Regression	XGBoost	Neural Network	Random Forest
Score 1	0.7687	0.9997	0.9971	0.9993
Score 2	0.7796	0.9998	0.9956	0.9998
Score 3	0.7623	0.9997	0.9978	0.9998
Score 4	0.7707	0.9998	0.9970	0.9998
Score 5	0.7745	0.9996	0.9986	0.9994
Average CV Score	0.7721	0.9997	0.9972	0.9996

### 3.3 Interpretation

**FIGURE 3:**
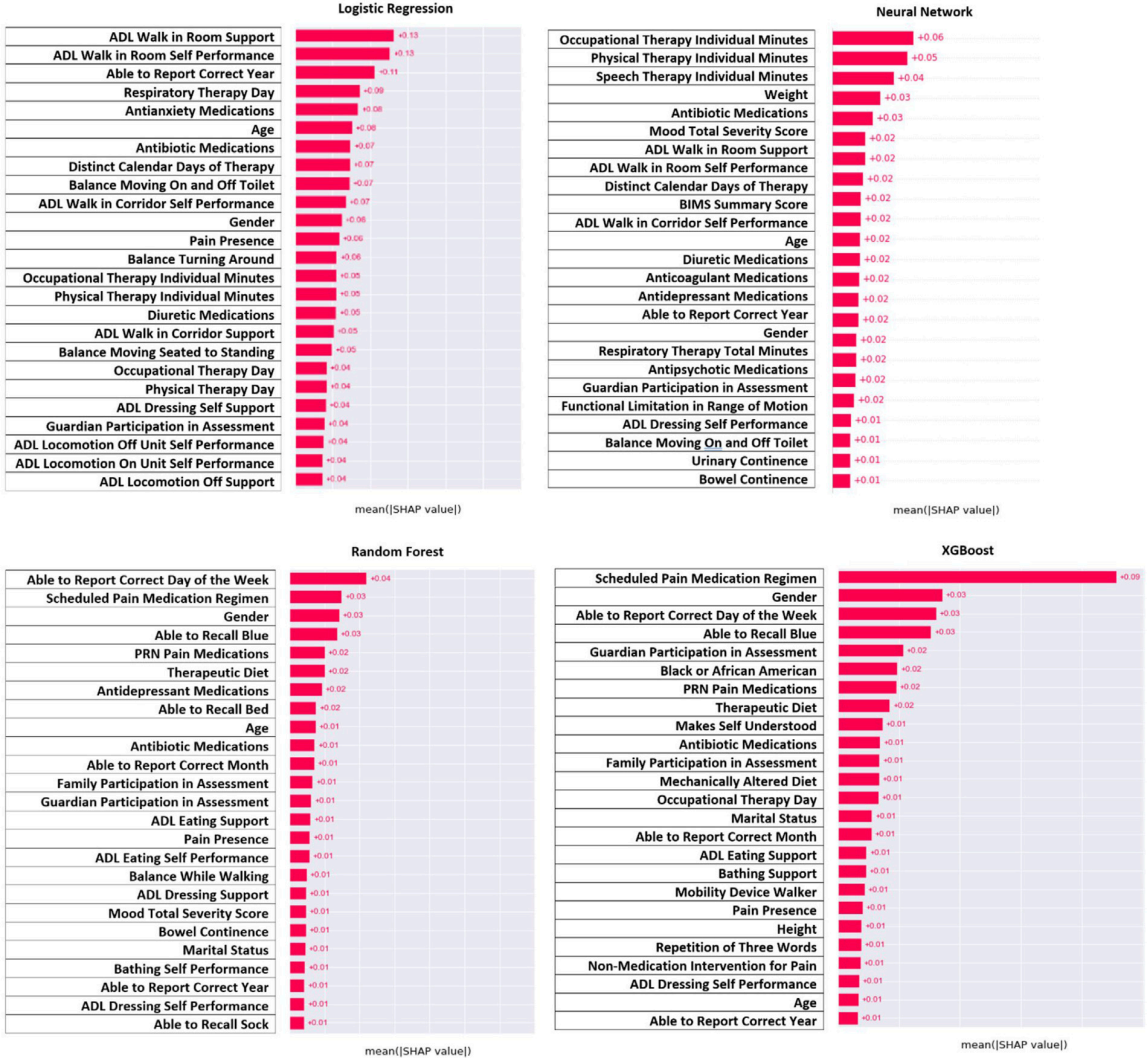
Shap values for top features to explain contribution to the model.

**FIGURE 4:**
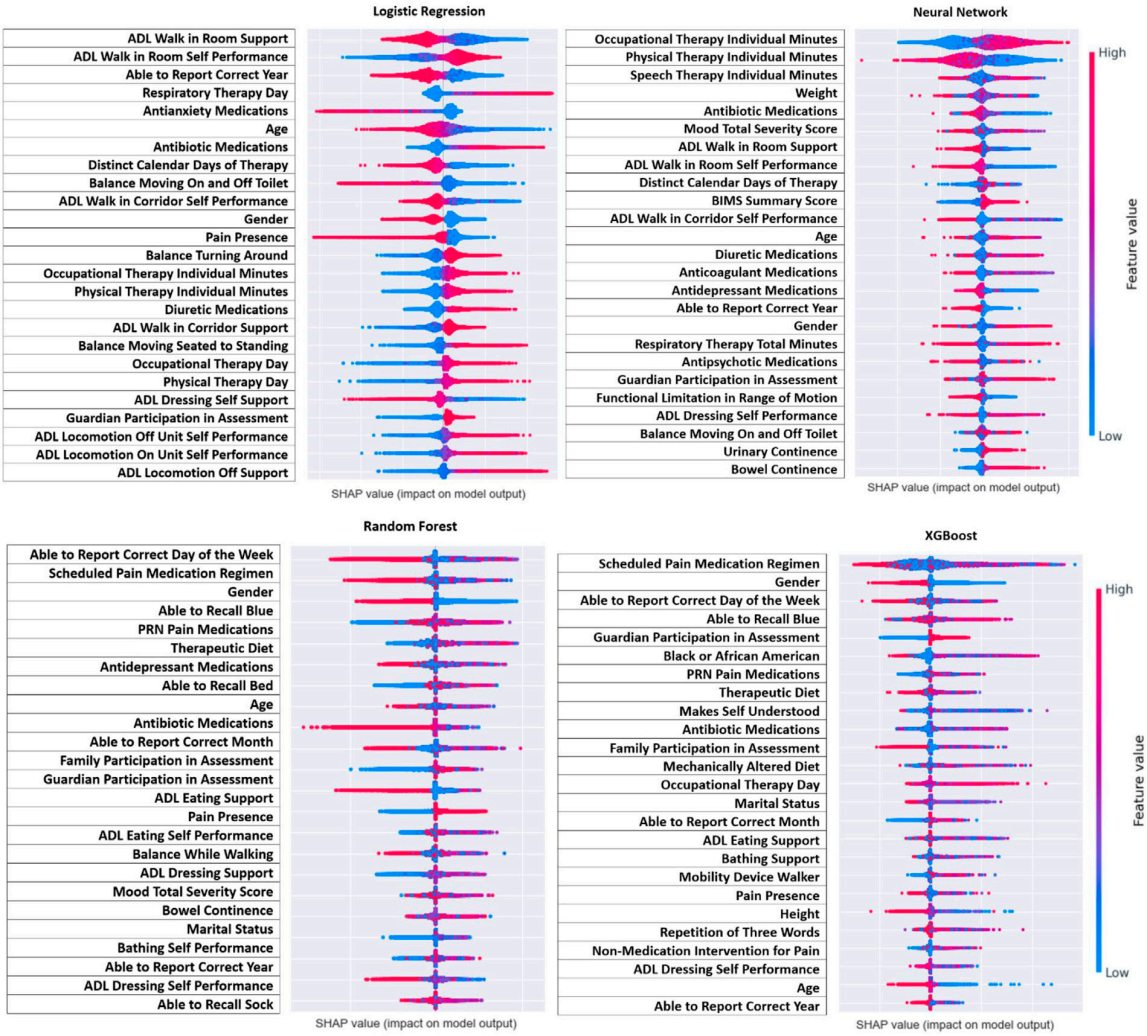
Direction of SHAP values for top features.

For model interpretation, the features that contributed the most to the model were those with the greatest absolute value of the SHAP values. Figure 3 demonstrates the features with the greatest contribution (absolute SHAP value) whereas Figure 4 demonstrates the direction of that contribution (positive or negative). It was important to interpret results from all four models since all models had a strong prediction ability, and comparison between models could identify similar features. Across all four models, it can be seen that the amount of therapy a person receives, their ability to be independent, and their overall mood contributed the most to predicting seizure following infection (Figure 3). For therapy, this was in the form of the number of minutes for speech, occupational, and physical therapy as well as the distinct calendar days and frequency of the therapy. For independence, this was in the form of activities for daily living for walking, mobility, eating, and dressing. Finally, mood was categorized based on mood severity score and the use of medications like antidepressants, antianxiety, and antipsychotics. Other notable features include antibiotic medications, diuretic medications, therapeutic diet, continence, and recall ability. Demographics also contributed to the model with age and gender being the most prominent.

Figure 4 demonstrates the direction of impact for each feature. Some features present in one model indicate the opposite effect in another model, or the direction is challenging to distinguish. However, across all four models it appears that residents who receive more therapy (speech, occupational, and physical), had lower ADL scores (more independent), and had a lower mood severity score (more positive mood), and took mood related medications (antidepressant or antianxiety) had lower risk of post-infection seizure. Lower age and male gender were associated with higher risk, but this was not as consistent across models as other findings.

## 4 Discussion

The models achieved a prediction accuracy between 76.5% and 99.9% for whether a stroke survivor will experience a seizure after an infection. It is plausible that data imputation and synthetic data created by up sampling artificially improved these metrics leading to an overly optimistic view of model performance. In other words, with balancing having a focus primarily on up sampling, the number of entries in the dataset was synthetically increased. These synthetic entries could have caused the dataset entries to become more distinct, making it easier to predict post-infection seizures. Up sampling also caused there to be more data and was thus more computationally intensive for future steps. However, up sampling is meant to reduce the bias of the majority class by up sampling the minority class, so the computational intensity is a tradeoff for reduced bias. Testing the model on larger, national populations would help to minimize adverse effects caused by up sampling and validate the resulting high accuracy. By focusing on all four models and their interpretation, the goal is to make the results more generalizable to future nursing home residents. The short-term contribution of the model is the use of SHAP values which allow for model interpretation, furthering the understanding of the relative importance of risk factors. Understanding feature importance through the SHAP values can guide the development of strategies to mitigate the effect of seizure risk for high-risk stroke survivors experiencing an infection.

The three main types of features that contributed the most to predictions were therapy, independence in locomotion and activities of daily living, and overall mood. The features best used to interpret the model are those related to therapy minutes (speech, occupational, and physical), distinct calendar days of therapy, the independence score for activities of daily living, mood severity score, use of antidepressant medications, use of antianxiety medications, and use of antipsychotic medications. The SHAP values indicated that stroke survivors who received more therapy, were able to be more independent, and had a better overall mood were at a lower risk of seizure following an infection. These results align with literature that has suggested that adults with epilepsy who exercise regularly reduce their risk of seizures (Nakken et al., 1990; Mario Arida et al., 2010). This is also true of adults with epilepsy who remain in a better mood and experience less stress to reduce their seizure risk (Jackson and Turkington, 2005; Sawyer and Escayg, 2010; McKee and Privitera, 2016). Regarding independence in stroke survivors, a decrease in independence leads to decreased mood during the recovery process (Albanese et al., 2020). This could indicate that as stroke survivors recover and become more independent, they would improve their mood and subsequently reduce their seizure risk. Although stroke survivors are not the same as people with epilepsy, stroke survivors still experience neurological complications thus have a risk of seizures. Identifying these features of therapy, independence, and mood allows healthcare providers and researchers potential levers for those residents at greater risk.

These features can be determined early, within the first 2 weeks of a resident’s admission to the SNF. When a resident enters a SNF, a therapy plan is set in place including the number of minutes of therapy they will receive each week. As time goes on, this plan will be updated to reflect their treatment needs, but from their admission assessment, physicians can estimate resident risk based on the number of minutes in the plan. Additionally, the resident receives scores for their mood severity and their independence during their first 14-day assessment. This would mean that residents who begin to show signs of less positive mood (have a high mood severity score), are more dependent (higher ADL scores), and receiving less therapy would be categorized as high risk. Providers could then identify these patients and determine if additional care is appropriate. Ultimately, the decision relies on the provider to take action to improve resident care, but this study helps contribute to the field of known risk factors. However, as this study is correlational and the studied population often have complex multi-morbid conditions, it is difficult to know whether the occurrence of therapy reduces the risk of seizure or if individuals with a risk of seizure are less able to receive therapy, or perhaps both. Disentangling this relationship will better inform resident care.

Other features that meaningfully contribute are those for antibiotic medications, antianxiety medications, PRN (pro re nata) pain medications, diuretic medications, gender, and therapeutic diet. Antibiotics are a less useful contribution since the use of antibiotics indicates an infection, which was already pre-established. Antianxiety, pain, and diuretic medications could once again be indicative of patient severity, but they could also indicate the possible presence of acute drug intoxication. Drug intoxication from antidepressants and pain medications has been found to cause seizures in patients with epilepsy (Chen et al., 2016), and it is possible that stroke survivors in SNF could also suffer from the same outcome. For the demographic factors, gender was previously discussed with the finding that males had a greater proportion of post infection seizure, and gender is also shown as a top contributing feature across models. Therapeutic diet was another feature found to contribute and represent the resident receiving altered meals to promote recovery. This feature likely represents a modifiable factor since lifestyle changes like diet tend to be important for promoting resident wellbeing.

In a simple reading of the results, if a resident is high risk, healthcare providers could enroll the resident in additional therapy time, encourage more independence, and focus on improving mood to reduce seizure risk. However, it is imperative to note that many important features may be signals of the severity of the resident’s condition rather than levers that can be pulled to improve their condition. For example, the results show that more therapy minutes are associated with reduced risk of seizure following infection. However, it may be the case that residents who are physically able to have therapy have less severe complications following their stroke. The severity of a resident’s condition following the stroke may be the influential factor underlying both the number of therapy minutes and the likelihood of seizure. Nevertheless, identifying these associations is valuable in furthering the discussion around improving post-stroke care in SNFs. That the model establishes associations rather than causal relationships should be considered as a limitation.

Aside from the resident post-stroke severity limitation, another limitation of the model is the way in which dates of the strokes, infections, and seizures are established. For determining the date of the event occurrence, the assessment date was used (or the date in which the SNF filled out the MDS data form). This is typically the practice for diagnosis dates for the MDS (Hua et al., 2021). However, it is likely that there is a lag between the time the event occurred and the assessment date. This means that a seizure could have occurred days prior but was not noted in the MDS until the date of the assessment. Therefore, the assumption was made that the lag time for all events to assessment was approximately the same. This would mean that the exact date of the event occurrence was not accurate, but the relation of the events to one another would be reasonably accurate. This assumption can be validated by the events in the MDS being chronological for individuals, such that the relation of events to one another is accurate (Mor et al., 2011). Thus, the time thresholds of 75 days between stroke and infection and 14 days between infection and seizure would be chronologically accurate with the exact time difference having some undetermined degree of error.

Similar to this relation between dates, there is also the possibility of error carried forward between assessments. For example, it was seen in the dataset that once a resident experienced a seizure, it often appeared in later consecutive assessments. This was caused by SNF staff using previous assessment data instead of reassessing the resident each time. The reason for this can be a variety of factors like understaffing, overcrowding, and distraction that cause staff to try to save time in completing assessments (Bowman, 2013). This made it challenging to distinguish repeated infections that then led to seizures in individual residents. As a result, unique residents were used for modeling, meaning that the residents could only have the stroke to infection to seizure pathway once. This caused the risk ratio to potentially indicate a smaller risk than if occurrences were evaluated. Additionally, this could have compromised some prediction and interpretation ability of the model. By only looking at one occurrence for each resident, it is possible that risk factors that contributed to a second or third occurrence would have been overlooked. This may also be the reason the model predicted so accurately if a first occurrence was easier to predict than subsequent occurrences. Although using unique residents may underestimate the risk ratio and miss risk factors in later occurrences, prevention of false post-infection seizures from error carried forward is more important. Risk factors can still be obtained by looking at unique residents, but using false post-infection seizures could skew results.

Even with the limitations of the model, it still serves as an effective tool for interpretation. Infections were associated with an increase in the risk of a seizure in stroke survivors through the calculation of a risk ratio and in the predictive model. The interpretability aspect of this model with SHAP allowed for the main factors that contribute to risk to be identified. These main factors that contribute to risk can help guide resident care. Looking into the future, this model and others in this research space could eventually be established to run in the background to continuously assess resident risk. Currently, the prediction aspect is not at the desired level for implementation into care, but with more iterations, the technology could eventually reach a high level. Hence these goals would be more suitable for long-term progress across the entire healthcare research field rather than individual study improvement. More realistically, implementation of the models on other datasets to confirm model performance and evaluate generalizability would be a more obtainable short-term goal. For an even longer-term goal bordering science fiction, having risk assessment across all resident diagnoses and all outcomes would be the greatest improvement for healthcare. For now, this individual resident risk and the interpretability of the model can help guide resident treatment to generate better outcomes for stroke survivors.

## 5 Conclusion

This machine learning model demonstrated a high degree of accuracy in predicting the occurrence of a seizure within 14 days following an infection in the population of stroke survivors at skilled nursing facilities. The interpretability of the model allowed for specific therapy, independence, and mood related features to be identified that are associated with the risk of seizure occurrence. This interpretability of the model can be used by healthcare providers to guide treatment decisions to prevent seizures in residents who suffered an infection.

## Data Availability

The data analyzed in this study is subject to the following licenses/restrictions: Data Sharing Agreement. Requests to access these datasets should be directed to nkong@purdue.edu.

## References

[B1] Ahmad F. B. Anderson R. N. (2021). The leading causes of death in the US for 2020. JAMA 325 (18), 1829–1830. 10.1001/jama.2021.5469 33787821 PMC8145781

[B2] Albanese A. Bartz-Overman C. Parikh T. Thielke S. (2020). Associations between activities of daily living independence and mental health status among Medicare managed care patients. J. Am. Geriatr. Soc. 68 (6), 1301–1306. 10.1111/jgs.16423 32196634

[B3] Bernhardt J. Churilov L. Ellery F. Collier J. Chamberlain J. Langhorne P. (2016). Prespecified dose-response analysis for A very early rehabilitation trial (AVERT). Neurology 86 (23), 2138–2145. 10.1212/WNL.0000000000002459 26888985 PMC4898313

[B4] Bindawas S. M. Vennu V. S. (2016). Stroke rehabilitation. A call to action in Saudi Arabia. Neurosci. J. 21 (4), 297–305. 10.17712/nsj.2016.4.20160075 PMC5224426 27744457

[B5] Bowman S. (2013). Impact of electronic health record systems on information integrity: quality and safety implications. Perspect. Health Inf. Manag. 10, 1c. PMC3797550 24159271

[B6] Bunney G. Murphy J. Colton K. Wang H. Shin H. J. Faigle R. (2022). Predicting early seizures after intracerebral hemorrhage with machine learning. Neurocrit Care 37, 322–327. 10.1007/s12028-022-01470-x 35288860 PMC10084721

[B7] CDC (2023) Stroke facts. United States: Centers for Disease Control and Prevention.

[B8] Chen C. Heinemann A. Granger C. Linn R. (2002). Functional gains and therapy intensity during subacute rehabilitation: a study of 20 facilities. Arch. Phys. Med. Rehabil. 83 (11), 1514–1523. 10.1053/apmr.2002.35107 12422318

[B9] Chen H. Y. Albertson T. E. Olson K. R. (2016). Treatment of drug-induced seizures. Br. J. Clin. Pharmacol. 81 (3), 412–419. 10.1111/bcp.12720 26174744 PMC4767205

[B10] Chohan S. A. Venkatesh P. K. How C. H. (2019). Long-term complications of stroke and secondary prevention: an overview for Primary Care Physicians. Singap. Med. J. 60 (12), 616–620. 10.11622/smedj.2019158 PMC7911065 31889205

[B11] Finlayson O. Kapral M. Hall R. Asllani E. Selchen D. Saposnik G. (2011). Risk factors, inpatient care, and outcomes of pneumonia after ischemic stroke. Neurology 77 (14), 1338–1345. 10.1212/WNL.0b013e31823152b1 21940613

[B12] Gittins M. Lugo-Palacios D. Vail A. Bowen A. Paley L. Bray B. (2020). Stroke impairment categories: a new way to classify the effects of stroke based on stroke-related impairments. Clin. Rehabil. 35 (3), 446–458. 10.1177/0269215520966473 33131321 PMC7944424

[B13] Graham L. (2013). Organization of rehabilitation services. Handb. Clin. Neurol. 110, 113–120. 10.1016/B978-0-444-52901-5.00010-1 23312635

[B14] Heron M. (2021). Deaths: leading causes for 2019. Natl. Vital Stat. 70 (9), 1–114. 34520342

[B15] Hua C. Thomas K. Bunker J. Gozalo P. Bélanger E. Mitchell S. (2021). Dementia diagnosis in the hospital and outcomes among patients with advanced dementia documented in the Minimum Data Set. J. Am. Geriatr. Soc. 70 (3), 846–853. 10.1111/jgs.17564 34797565 PMC8904279

[B16] IHCP (2023) Indiana health coverage programs: long-term care. Indiana Family and Social Services Administration.

[B17] Jackson M. Turkington D. (2005). Depression and anxiety in epilepsy. J. Neurol. Neurosurg. Psychiatry 76 (1), i45–i47. 10.1136/jnnp.2004.060467 15718221 PMC1765680

[B18] Jäger S. Allhorn A. Bießmann F. (2021). A benchmark for data imputation methods. Front. Big Data. 4, 693674. 10.3389/fdata.2021.693674 34308343 PMC8297389

[B19] Jette D. Warren R. Wirtalla C. (2004). Rehabilitation in skilled nursing facilities: effect of nursing staff level and therapy intensity on outcomes. Am. J. Phys. Med. Rehabil. 83 (9), 704–712. 10.1097/01.phm.0000137312.06545.d0 15314535

[B20] Jette D. Warren R. Wirtalla C. (2005). The relation between therapy intensity and outcomes of rehabilitation in skilled nursing facilities. Arch. Phys. Med. Rehabil. 86 (3), 373–379. 10.1016/j.apmr.2004.10.018 15759214

[B21] Jung H.-Y. Trivedi A. Grabowski D. Mor V. (2016). Does more therapy in skilled nursing facilities lead to better outcomes in patients with hip fracture? Phys. Ther. 96 (1), 81–89. 10.2522/ptj.20150090 26586858 PMC4706596

[B22] Kelly-Hayes M. Robertson J. Broderick J. Duncan P. Hershey L. Roth E. (1998). The American heart association stroke outcome classification: executive summary. Circulation 97 (24), 2474–2478. 10.1161/01.cir.97.24.2474 9641702

[B23] Koopmans R. Lavrijsen J. Hoek F. Went P. Schols J. (2010). Dutch elderly care physician: a new generation of nursing home physician specialists. J. Am. Geriatr. Soc. 58 (9), 1807–1809. 10.1111/j.1532-5415.2010.03043.x 20863347

[B24] Langhorne P. Stott D. Robertson L. MacDonald J. Jones L. McAlpine C. (2000). Medical complications after stroke: a multicenter study. AHA J. 31, 1223–1229. 10.1161/01.str.31.6.1223 10835436

[B25] Learoyd A. Woodhouse L. Shaw L. Sprigg N. Bereczki D. Berge E. (2017). Infections up to 76 days after stroke increase disability and Death. Transl. Stroke Res. 8 (6), 541–548. 10.1007/s12975-017-0553-3 28752410 PMC5818141

[B26] Lekoubou A. Petucci J. Ajala T. F. Katoch A. Sen S. Honavar V. (2024) Large datasets from Electronic Health Records predict seizures after ischemic strokes: a Machine Learning approach. medRxiv. 10.1101/2024.01.24.24301755

[B27] Lenze E. Host H. Hildebrand M. Morrow-Howell N. Carpenter B. Freedland K. (2012). Enhanced medical rehabilitation increases therapy intensity and engagement and improves functional outcomes in postacute rehabilitation of older adults: a randomized-controlled trial. J. Am. Med. Dir. Asso 13 (8), 708–712. 10.1016/j.jamda.2012.06.014 PMC3601780 22863663

[B28] Levinson D. (2013). Skilled nursing facilities often fail to meet care planning and discharge planning requirements. Office of Inspector General. 10.1016/j.gerinurse.2013.04.005 23639913

[B29] Looti A. Petucci J. Katoch A. Honavar V. (2023). Machine learning prediction of seizures after ischemic strokes. (S30.007). Neurology 100 (17). 10.1212/WNL.0000000000203063

[B30] Mario Arida R. Alexandre Scorza F. Gomes da Silva S. Schachter S. Abrão Cavalheiro E. (2010). The potential role of physical exercise in the treatment of epilepsy. Epilepsy Behav. 17 (4), 432–435. 10.1016/j.yebeh.2010.01.013 20159660

[B31] McKee H. Privitera M. (2016). Stress as a seizure precipitant: identification, associated factors, and treatment options. Seizure 44, 21–26. 10.1016/j.seizure.2016.12.009 28063791

[B32] Mor V. Intrator O. Unruh M. A. Cai S. (2011). Temporal and geographic variation in the validity and internal consistency of the nursing home resident assessment Minimum data set 2.0. BMC Health Serv. Res. 11, 78. 10.1186/1472-6963-11-78 21496257 PMC3097253

[B33] Myint P. Staufenberg E. Sabanathan K. (2006). Post-stroke seizure and post-stroke epilepsy. Postgrad. Med. J. 82 (971), 568–572. 10.1136/pgmj.2005.041426 16954451 PMC2585721

[B34] Naess H. Nyland H. Thomassen L. Aarseth J. Myhr K. (2004). Long-term outcome of cerebral infarction in young adults. Acta Neurol. Scand. 110 (2), 107–112. 10.1111/j.1600-0404.2004.00273.x 15242418

[B35] Nakken K. Bjørholt P. Johannessen S. LoSyning T. Lind E. (1990). Effect of physical training on aerobic capacity, seizure occurrence, and serum level of antiepileptic drugs in adults with epilepsy. Epilepsia 31 (1), 88–94. 10.1111/j.1528-1157.1990.tb05365.x 2303017

[B36] Salgado C. M. Azevedo C. Proença H. Vieira S. M. (2016) Secondary analysis of electronic health records. Cham: Springer.

[B37] Sawyer N. Escayg A. (2010). Stress and epilepsy: multiple models, multiple outcomes. J. Clin. Neurophysiol. 27 (6), 445–452. 10.1097/WNP.0b013e3181fe0573 21076337

[B38] Shiels M. S. Haque A. T. González A. B. Freedman N. D. (2022). Leading causes of death in the US during the COVID-19 pandemic, march 2020 to october 2021. JAMA Intern. Med. 182 (8), 883–886. 10.1001/jamainternmed.2022.2476 35788262 PMC9257676

[B39] Smajlović D. Kojić B. Sinanović O. (2006). Five-year survival after first-ever stroke. Bosn. J. Basic Med. Sci. 6 (3), 17–22. 10.17305/bjbms.2006.3138 16995842 PMC7193668

[B40] Ulm L. Harms H. Ohlraun S. Reimnitz P. Meisel A. (2012). Impact of infections on long-term outcome after severe middle cerebral artery infarction. J. Neurol. Sci. 319 (1-2), 15–17. 10.1016/j.jns.2012.05.042 22682764

[B41] Vezzani A. Fujinami R. White H. S. Preux P.-M. Blümcke I. Sander J. (2015). Infections, inflammation and epilepsy. Acta Neuropathol. 131 (2), 211–234. 10.1007/s00401-015-1481-5 26423537 PMC4867498

[B42] Wells B. J. Chagin K. M. Nowacki A. S. Kattan M. W. (2013). Strategies for handling missing data in electronic health record derived data. EGEMS (Wash DC). 1 (3), 1035. 10.13063/2327-9214.1035 25848578 PMC4371484

